# The Sigma-1 Receptor Agonist Fluvoxamine Is Protective in Hyperglycaemia-Induced Dysfunction of Trabecular Meshwork Cells

**DOI:** 10.3390/ph19030385

**Published:** 2026-02-27

**Authors:** Alexandra Rozsahegyi, Marcell Cserhalmi, Timea Medveczki, Zsuzsanna Buzogany, Eva Ruisanchez, Andras Budai, Balazs Besztercei, Attila J Szabo, Judit Hodrea, Andrea Fekete

**Affiliations:** 1 MTA-SE Lendület “Momentum” Diabetes Research Group, 1083 Budapest, Hungary; rozsahegyi.alexandra@phd.semmelweis.hu (A.R.); marcell.cserhalmi@gmail.com (M.C.); medveczki.timea@gmail.com (T.M.); buzogany.zsuzsanna@phd.semmelweis.hu (Z.B.); hodrea.judit@semmelweis.hu (J.H.); 2 Pediatric Center, MTA Center of Excellence, Semmelweis University, 1083 Budapest, Hungary; szabo.attila@semmelweis.hu; 3 Institute of Translational Medicine, Semmelweis University, 1094 Budapest, Hungary; ruisanchez.eva@semmelweis.hu (E.R.); besztercei.balazs@semmelweis.hu (B.B.); 4 HUN-REN-SU Cerebrovascular and Neurocognitive Disorders Research Group, 1094 Budapest, Hungary; 5 Department of Pathology, Forensic and Insurance Medicine, Semmelweis University, 1091 Budapest, Hungary; budai.andras@semmelweis.hu

**Keywords:** Sigma-1 receptor, trabecular meshwork, fibrosis, diabetes mellitus, hyperglycaemia, oxidative stress, fluvoxamine, Wistar rat, db/db mice, HTM5

## Abstract

**Background/Objectives**: Diabetes mellitus (DM) is associated with a doubled prevalence of elevated intraocular pressure (IOP) caused by trabecular meshwork (TM) dysfunction. Chronic hyperglycaemia leads to oxidative stress and fibrotic remodeling of the TM. We previously identified the Sigma-1 receptor (S1R) as a novel anti-fibrotic target by demonstrating that its agonist, fluvoxamine (FLU), is protective in diabetes-related renal fibrosis. Here, we investigate its potential to mitigate ocular fibrosis. **Methods**: First, we wanted to verify in different in vivo models (high-fat diet/streptozotocin (HFD/STZ) rats, db/db mice) that type 2 DM (T2DM) leads to fibrotic remodeling of the TM. Then, in vitro, we assessed the effect of FLU (15 µM) on hyperglycaemia-induced (HG, 25 µM) fibrosis, oxidative stress and endogenous nitric oxide (NO) production. **Results**: In T2DM models, excessive accumulation of collagen, α-smooth muscle actin (αSMA), fibronectin (Fn) and F-actin was observed in the eyes. Ocular fibrosis was accompanied by IOP elevation (13.7 vs. 18.7 mmHg) in db/db mice. In human TM cells (HTM5), FLU decreased HG-induced cell proliferation (14% vs. 24%) and upregulated S1R protein expression. Furthermore, FLU suppressed the expressions of key fibrotic elements, including transforming growth factor-β2 (*TGF-β2*) by 37%, Fn by 49%, collagen type 1 (*COL1A1*) and type 4 (COL4A1) by 24% and 45%, respectively. FLU also reversed HG-induced F-actin accumulation by 39% and enhanced intracellular NO levels by 34%. Crucially, FLU decreased ROS generation by half, demonstrating its protective effect against HG-induced oxidative stress. **Conclusions**: These findings highlight the potential of S1R activation as a promising therapeutic target to alleviate hyperglycaemia-induced injury to the TM by modulating multiple molecular pathways.

## 1. Introduction

Diabetes mellitus (DM)-associated ocular complications, including retinopathy, are the leading cause of irreversible vision loss worldwide. Thirty percent of diabetic patients have some form of eye disease, and chronic hyperglycaemia roughly doubles the risk of glaucoma and elevated intraocular pressure (IOP) [1,2,3,4,5,6,7,8,9].

Glucose concentration in the aqueous humor (AH) of diabetic patients is higher than in non-diabetic controls and correlates with HbA1C levels [10]. This may be directly linked to increased IOP and glaucoma, by promoting fibrotic transformation, extracellular matrix (ECM) accumulation [11], and triggering oxidative and nitrosative stress [12,13,14] in the trabecular meshwork (TM), the primary drainage pathway for AH. Fibrotic remodeling within the TM includes signaling cascades regulated by key mediators [15,16,17,18], such as transforming growth factor-β2 (TGF-β2). Its upregulation drives ECM deposition, restricting AH outflow and contributing to an increase in IOP [19], which damages retinal ganglion cells and leads to progressive vision loss [20,21,22].

Despite the central role of TM fibrosis, current glaucoma treatments, such as prostaglandine analogues or beta-blockers, do not directly modify fibrotic remodeling [23]. This persistent therapeutic gap highlights the need for novel anti-fibrotic strategies directly targeting TM function.

Sigma-1 receptor (S1R) is a transmembrane chaperone protein that serves as a multifunctional therapeutic target due to its neuroprotective, anti-inflammatory, and antioxidant effects [24,25]. In the eye, S1R is localized in the lacrimal gland [26], the iris-ciliary body [27], the lens [28], the retinal tissue [29,30], and, as we demonstrated for the first time, also in the TM [31]. By regulating different molecular pathways, S1R activation mitigates endoplasmic reticulum (ER) stress [32,33,34], inflammation [35,36], and preserves redox homeostasis [37,38]. Our group, for the first time, demonstrated that S1R activation is antifibrotic across various tissues, including the diabetic kidney, by modulating ER stress and oxidative pathways [16,39,40]. Furthermore, we demonstrated that S1R agonists prevent TGF-β2-induced fibrosis in the human TM [16].

Based on these results, here we first wanted to verify in different in vivo models that type 2 DM (T2DM) leads to fibrotic remodeling of the TM. Then, in vitro, we assessed the effect of the S1R agonist fluvoxamine (FLU) on hyperglycaemia-induced fibrosis and dysfunction in human TM cells. We hypothesize that S1R agonists can alleviate hyperglycaemia-induced TM injury and may have promising therapeutic potential in diabetes-associated ocular complications.

## 2. Results

### 2.1. Diabetes Induces IOP Elevation and Cytoskeletal Rearrangement of TM in db/db Mice

**Figure 1:**
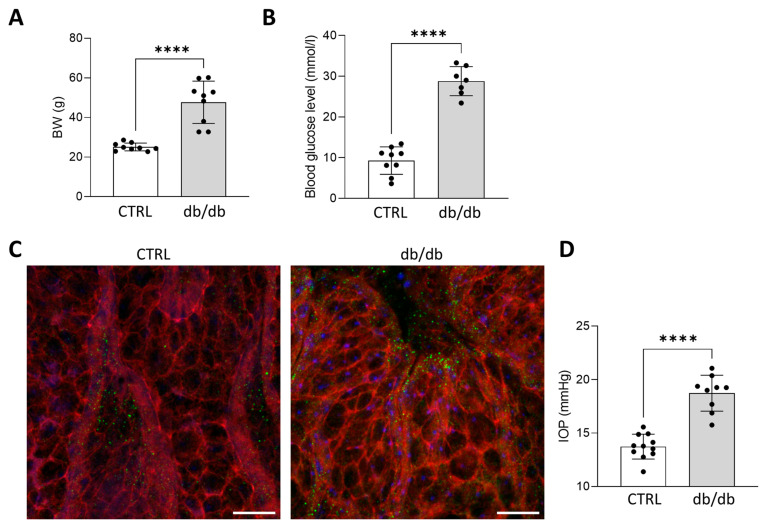
(**A**) Final bodyweight (BW) and (**B**) blood glucose levels of control (CTRL) and diabetic (db/db) mice. (**C**) Representative immunohistochemical images of the TM region of CTRL and db/db mice (F-actin: red, nuclei: blue, Sigma-1 receptor (S1R): green, objective: 20×, scale bar: 50 µm, Abberior Expert Line confocal microscope (Abberior Instruments, Göttingen, Germany)). (**D**) Intraocular pressure (IOP) in CTRL and db/db mice at the end of the experimental protocol. Bars indicate mean ± SD; *n* = 7–11/group; **** *p* < 0.0001.

T2DM was confirmed by increased blood glucose levels (CTRL: 9.2 ± 3.16 mmol/L vs. db/db: 28.8 ± 3.3 mmol/L, *p* < 0.0001) and higher bodyweights (Figure 1A,B) in db/db mice (CTRL: 25 ± 1.98 g vs. db/db: 48 ± 10.66 g, *p* < 0.0001) and S1R was expressed in the TM of both groups (Figure 1C). F-actin levels of the explanted TM visualized by phalloidin staining were increased in db/db mice, suggesting elevated tissue stiffness and increased resistance to AH outflow. Parallel with this, IOP was significantly higher (*p* < 0.0001) in db/db mice (CTRL: 13.7 ± 1.16 mmHg vs. db/db: 18.7 ± 1.68 mmHg, *p* < 0.0001) (Figure 1D). These results indicate that enhanced cellular contractility and pathological cytoskeletal remodeling directly contributed to IOP elevation.

### 2.2. Hyperglycaemia Induces Ocular Fibrosis in the Eyes of HFD/STZ Rats

**Figure 2:**
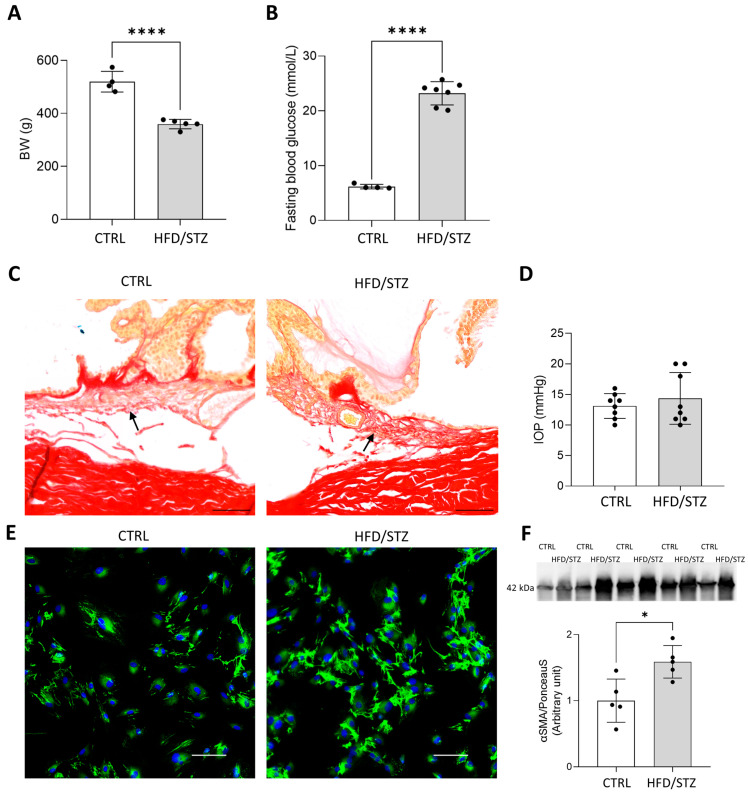
(**A**) BW and (**B**) fasting blood glucose levels of control (CTRL) and high-fat diet (HFD) + streptozotocin (STZ)-induced diabetic rats (HFD/STZ). (**C**) Representative histological images stained with picrosirius red of the TM tissue from CTRL and HFD/STZ rats (arrows point to the TM region, scale bar: 50 µm). (**D**) IOP in CTRL and HFD/STZ groups at the end of the experimental protocol. (**E**) Representative immunocytochemical images showing fibronectin (Fn) expression in primary rat TM (prTM) cells derived from CTRL and HFD/STZ rats (Fn: green, nuclei: blue; Nikon Eclipse Ti2 microscope (Nikon Instruments, Melville, NY, USA); objective: 20×; scale bar: 100 µm.) (**F**) Upper panel: Representative images of Western blot analysis of α-smooth muscle actin (αSMA, 43 kDa). Lower panel: Protein level of αSMA in prTM cell homogenates derived from CTRL and HFD/STZ rats. Bars indicate mean ± SD; *n* = 4–9/group; * *p* < 0.05; **** *p* < 0.0001.

To strengthen our findings and further characterize diabetes-induced TM fibrosis, an additional T2DM model in another species was introduced. T2DM was induced in Wistar rats by a high-fat diet (HFD) combined with streptozotocin (STZ; HFD/STZ rats). Final bodyweight was lower (CTRL: 519 ± 39 g vs. HFD/STZ: 359 ± 17 g, *p* < 0.0001, Figure 2A) and fasting blood glucose levels were increased in HFD/STZ rats (CTRL: 6.2 ± 0.4 mmol/L vs. HFD/STZ: 23.2 ± 2.1 mmol/L, *p* < 0.0001, Figure 2B).

The overall structure of TM was preserved in both groups, while in the TM regions of HFD/STZ rats, higher collagen accumulation was detected (Figure 2C). In parallel, more intense fibronectin (Fn) staining (Figure 2E) and increased level of α-smooth muscle actin (αSMA; *p* < 0.05) (Figure 2F) were measured in prTM cells isolated from HFD/STZ rats, suggesting the development of TM fibrosis.

Interestingly enough, IOP was the same in both groups despite the significant fibrotic changes (Figure 2D).

### 2.3. S1R Is Decreased in Diabetic TM and FLU Elevates Its Level

**Figure 3:**
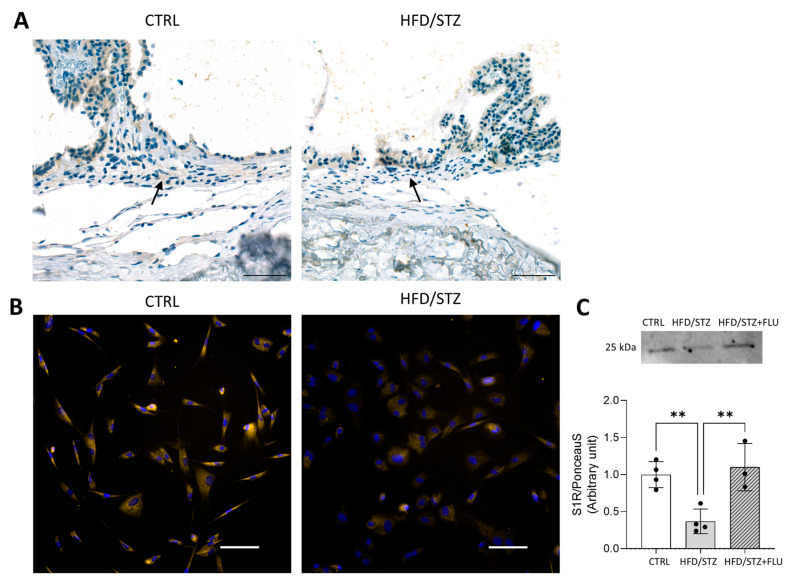
(**A**) Representative histological images of S1R staining in the TM tissue from CTRL and HFD/STZ rats (arrows point to the TM; S1R: brown, nuclei: blue; Panoramic1000 slide scanner (3DHISTECH, Budapest, Hungary), scale bar: 50 µm). (**B**) Representative immunocytochemical images of S1R in prTM derived from CTRL and HFD/STZ rats (S1R: yellow, nuclei: blue; Nikon Eclipse Ti2 microscope, objective: 20×; scale bar: 100 µm). (**C**) Western blot analysis of S1R level (25 kDa) in prTM cells isolated from CTRL and HFD/STZ animals, and after 48 h fluvoxamine (FLU, 15 µM) treatment of the cells derived from the diabetic group (HFD/STZ + FLU). Bars indicate mean ± SD; *n* = 3–4/group; ** *p* < 0.01.

Histological staining confirmed that S1R is endogenously expressed in the TM tissue of CTRL and HFD/STZ rats, with reduced S1R protein levels in the diabetic tissue (Figure 3A). This downregulation was further confirmed in prTM cells isolated from the animals, which exhibited reduced S1R expression measured by immunocytochemistry (ICC) and Western blot (Figure 3B,C). Treatment of the cells with FLU significantly elevated the S1R protein level.

### 2.4. FLU Increases S1R Protein Level in HTM5 Cells

The function of the S1R remains largely unexplored, particularly in the anterior segment, including the diabetic TM. We previously showed for the first time that S1R is expressed in HTM5 cells [39], and here, we investigated the effects of high glucose (HG, 25 mM) and FLU treatment on S1R levels and subcellular localization.

**Figure 4:**
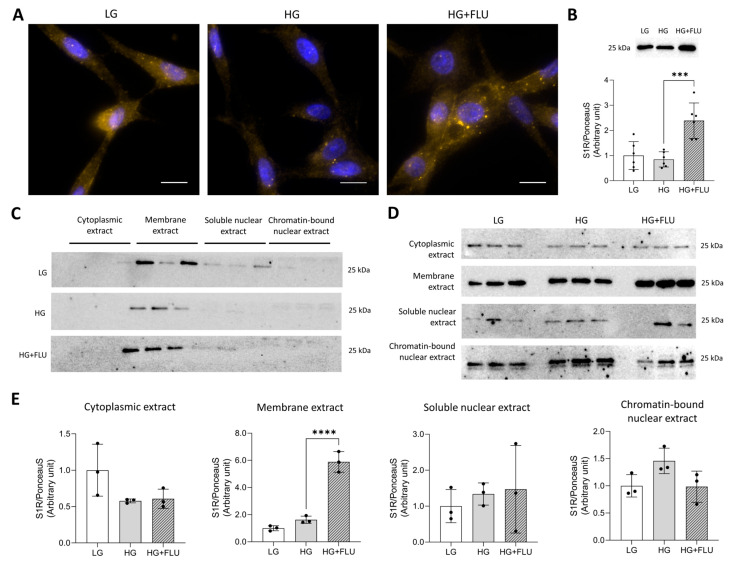
(**A**) Representative images of S1R in human TM (HTM5) cells treated with high glucose (HG, 25 mM) or HG + 15 µM fluvoxamine (HG + FLU) (S1R: yellow, nuclei: blue; Nikon Eclipse Ti2 microscope, objective: 60×, scale bar: 10 µm). (**B**) Upper panel: Representative images of Western blot, lower panel: S1R protein levels in total homogenates of HTM5 cells (data: mean ± SD; *n* = 6/group; *** *p* < 0.001). (**C**) Representative images of Western blot of S1R protein level in different subcellular fractions of HTM5 cells. Each fraction was analyzed on an individual blot to compare the distribution of S1R across the four fractions under the three experimental conditions (LG, HG, HG + FLU). (**D**,**E**). In a complementary setup, samples from the three treatments were loaded on the same blot to compare S1R protein levels within each extract. Bars indicate mean ± SD; *n* = 3/group, **** *p* < 0.0001.

HG exposure did not alter S1R levels; however, FLU treatment led to a significant S1R upregulation (*p* < 0.0001; Figure 4A,B). S1R was mainly detected in the membrane fraction under all experimental conditions (Figure 4C). In the comparative setup, FLU increased S1R levels in the membrane fraction, while no changes were observed in any of the other subcellular compartments (Figure 4D,E).

### 2.5. S1R Agonist FLU Reduces HG-Induced Cell Proliferation and Fibrotic Response in HTM5 Cells

Higher AH outflow resistance results from fibrotic remodelling and ECM accumulation in the TM. In the AH of diabetic patients, glucose concentration is elevated; therefore, here we assessed whether HG exposure affects the proliferative and fibrotic responses of HTM5 cells, and how S1R activation influences these changes.

**Figure 5:**
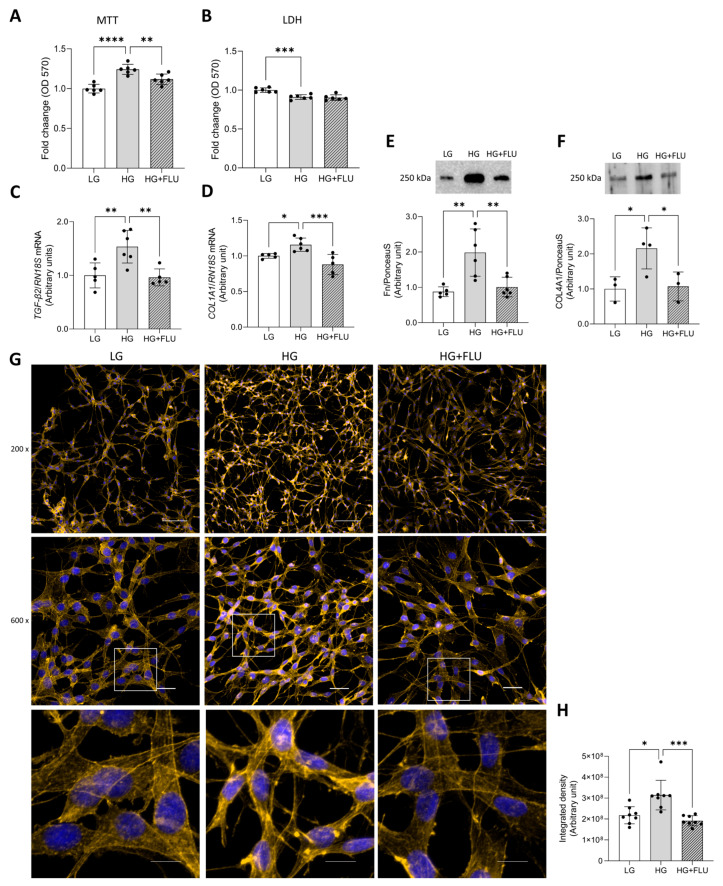
Effects of 15 µM FLU (HG + FLU) treatment on HG–induced changes in HTM5 cells: methyl-thiazolyl diphenyl-tetrazolium bromide (MTT) assay was performed to measure cell proliferation (**A**) and lactate dehydrogenase (LDH) assay to asses cytotoxicity (**B**), mRNA expression of transforming growth factor β2 (*TGF-β2*) (**C**), collagen type 1 (*COL1A1*) (**D**), protein levels of fibronectin (Fn) (**E**), collagen type 4 (COL4A1) (**F**), and ICC staining of F-actin (**G**) and the calculated integrated density of the ICC images (**H**) (F-actin: yellow; nuclei: blue; Nikon Eclipse Ti2 microscope; objectives: 20×; 60×, cropped images: 600 × 600 pixels; scale bars: 100 μm, 20 μm, 10 μm respectively). Bars indicate mean ± SD, *n* = 3–8/groups, * *p* < 0.05; ** *p* < 0.01; *** *p* < 0.001; **** *p* < 0.0001.

Exposure to HG for 48 h increased cell proliferation, and FLU treatment reduced this hyperproliferative response. In contrast, lactate dehydrogenase (LDH) assay results indicated lower cytotoxicity under HG conditions compared to LG medium, suggesting enhanced cell viability (Figure 5A,B). Together, these findings imply that HG promotes cell survival and proliferation, and FLU mitigates this hyperproliferative effect without inducing cytotoxicity. HG elevated the mRNA expression of *TGF-β2* (Figure 5C) and collagen type 1 (*COL1A1*) (Figure 5D). It also increased protein levels of Fn (Figure 5E) and collagen type 4 (COL4A1) (Figure 5F). Furthermore, HG also induced F-actin reorganisation, actin clump and stress fiber formation (Figure 5G,H). FLU treatment markedly reduced all these changes, indicating the protective effect of S1R activation against fibrotic remodeling of the TM.

### 2.6. FLU Elevates Intracellular Nitric Oxide (NO) Levels and Reduces Endogenous Reactive Oxygen Species (ROS) in HTM5 Cells

**Figure 6:**
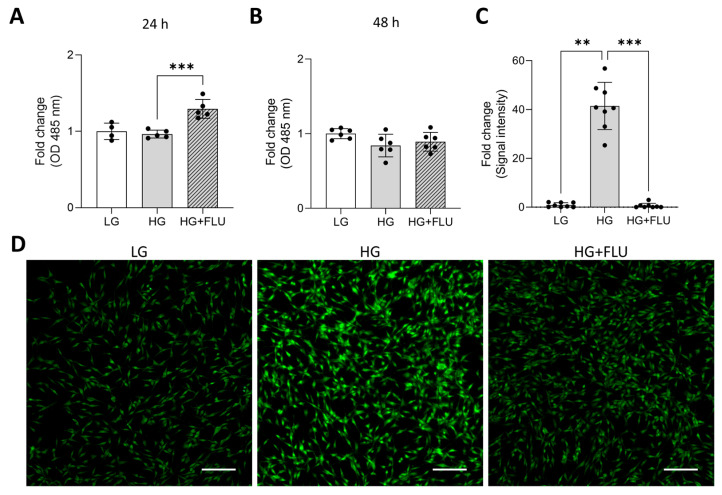
Measurement of intracellular nitric oxide (NO) level in HG-induced (HG) and FLU (15 µM)-treated (HG + FLU) HTM5 cells using DAF-FM fluorescence assay for 24 h (**A**) and 48 h (**B**). Fluorescent detection of reactive oxygen species (ROS) in HTM5 cells exposed to HG and treated with HG + FLU (**C**,**D**). Nikon Eclipse Ti2 microscope, objective: 20×; scale bar: 100 µm. Bars indicate mean ± SD; *n* = 3–8/group, ** *p* < 0.01; *** *p* < 0.001.

As a potent vasodilator, NO plays a crucial role in maintaining normal IOP by regulating contractility within the TM and controlling AH outflow resistance [41]. To determine whether S1R activation modulates NO generation in HG-induced HTM5 cells, we measured NO levels using the DAF-FM fluorescent probe. However, intracellular NO levels were not altered by HG exposure; FLU treatment increased NO production after 24 h of induction. NO level restored to control levels by 48 h, suggesting a time-dependent FLU effect (Figure 6A,B). This result indicated that S1R activation could facilitate TM relaxation by rapidly enhancing NO production and AH outflow in hyperglycaemic conditions.

Multiple studies have shown that oxidative stress, driven by accelerated ROS production, contributes to optic nerve degeneration and TM damage [42]. Semi-quantitative analysis of fluorescence images demonstrated marked elevation of ROS in HG-exposed HTM5 cells. FLU significantly reduced this accumulation, indicating the potential antioxidative effect of FLU under hyperglycaemic stress (Figure 6C,D).

## 3. Discussion

Ocular fibrosis, characterized by aberrant accumulation of ECM, is a grave pathological process that frequently leads to irreversible vision loss [43]. Its clinical relevance is high in the anterior chamber, where fibrosis of the TM increases resistance to AH outflow, thereby raising IOP. The elevated IOP causes subsequent damage to the optic nerve through the progressive apoptosis of retinal ganglion cells, ultimately resulting in visual deterioration [44].

DM is a major contributor to various ocular complications, promoting widespread organ dysfunction through the induction of oxidative stress, inflammation and cellular apoptosis. Previous studies demonstrated an association between high blood glucose and increased IOP [13], while others reported significantly higher glucose levels in the AH of diabetic patients [45]. Chronic hyperglycaemia can induce multiple stress-related pathways in the TM, besides oxidative stress and apoptosis, ECM remodelling and fibrosis develop, which are detrimental to ocular structures and increase AH outflow resistance and IOP [14].

In this study, our first objective was to confirm the core pathology by showing in vivo that hyperglycaemia leads to TM fibrosis in diabetic animals. Therefore, we used two distinct rodent models of T2DM. In db/db mice, parallel to elevated IOP, the level of F-actin was increased, a cytoskeletal change linked to tissue stiffening, and a characteristic cellular response to profibrotic signalling [46]. HFD/STZ-induced Wistar rat model also demonstrated excessive collagen deposition in the TM and a significant increase in Fn and αSMA levels. Interestingly, in this model, DM-induced fibrotic remodeling within the TM was not accompanied by increased IOP.

While a general association exists, the manifestation of IOP elevation varies across clinical reports and animal models [47]. Glaucoma is often, but not necessarily, accompanied by elevated IOP. It is seen that 30–90% of patients present with ‘normal’ IOP. In accordance with this, our results also suggest that the primary fibrotic changes and TM dysfunction can occur independently of ocular hypertension. The differential IOP response between models highlights the complex, multi-factorial pathogenesis and emphasises that TM fibrosis can be the early and primary site of injury, irrespective of IOP dynamics.

Once the development of TM fibrosis was verified in DM models, our second aim was to explore its pathology further and identify novel therapeutic options. Current treatments, including topical and systemic medications, primarily focus on reducing IOP by either decreasing AH production or increasing outflow [48], strategies that address the symptom rather than the core pathology. Furthermore, their efficacy is limited by a high incidence of local and systemic side effects, leading to poor patient adherence [49]. This collective unsatisfactory profile emphasizes the critical need for a new approach that moves beyond pressure management.

The S1R is recognized for its crucial role in cellular survival and homeostasis [50]. It is primarily expressed in the central nervous system, but lately it has also been described in peripheral organs such as the heart, the liver, the kidneys, and the eyes [51]. The S1R is well-documented for its widespread protective effect, although its function in the TM region remains largely unexplored. Previously, we first confirmed abundant S1R expression in HTM5 cells and showed that S1R primarily resides in the endoplasmic reticulum of TM cells [39]. Here, we extended these observations by demonstrating that S1R is differentially abundant in various intracellular compartments, and FLU increases its protein level only in the membrane extract. These data also suggest that S1R does not undergo major translocation upon FLU treatment. Hyperglycaemia, however, leads to decreased S1R levels, as we observed downregulation of the receptor in the ocular tissues of HFD/STZ rats. This observation correlates with a previous report showing that S1R levels were considerably decreased in the diabetic animal model and upon HG-induction [52].

Building on these results and the hypothesis that hyperglycaemia in DM directly suppresses S1R expression in ocular tissue, leading to TM fibrosis, we focused on the anti-fibrotic potential of S1R activation. We used FLU as a high-affinity agonist of the S1R [53,54]. Our aim was to evaluate its effects in HTM5 cells exposed to an HG environment to mimic diabetic conditions. First, we showed that FLU treatment restored the S1R levels and reduced HG-induced proliferation of HTM5 cells. Furthermore, FLU treatment significantly alleviated the fibrotic response by decreasing *TGF-β2*, Fn, *COL1A1* and COL4A1 levels and reducing F-actin reorganisation in HG-exposed cells. Our results strengthen previous findings that a hyperglycaemic environment induces fibrotic transformation [12,14] and suggest that S1R activation may be a potential therapeutic strategy for mitigating diabetes-induced TM fibrosis.

The current study found that FLU also significantly elevates NO levels under HG conditions. NO has gained attention as a novel therapeutic agent for glaucoma treatment, functioning as a key activator of soluble guanylate cyclase to modulate trabecular outflow. This process depends on the TM, given that its cellular components exhibit smooth muscle cell-like contractile activity [55], in which NO induces cell relaxation and cytoskeletal remodelling [56,57]. Our previous work demonstrated that activating the S1R with FLU resulted in increased NO production in HTM5 cells [39]. This NO restoration in a hyperglycaemic environment is highly relevant, as NO levels and its synthase expression are known to be reduced in diabetic conditions [58,59] and also in glaucoma [60,61].

S1R is also known to regulate the redox state and protect against ROS-mediated damage [38,62]. Consistent with this, our study demonstrated that FLU mitigates intracellular ROS generation in HTM5 cells exposed to HG conditions. This key finding confirms that S1R activation effectively reverses the oxidative burden imposed by the diabetic environment. This therapeutic potential is highly significant, as DM impairs the TM through persistent hyperglycaemia, which enhances mitochondrial ROS production, creating a highly profibrotic microenvironment [63,64]. Chen et al. demonstrated that the hyperglycaemic state directly influences HTM5 cell ROS production in a concentration-dependent manner [12], while Singh et al. showed activation and upregulation of genes that drive oxidative injury and profibrotic changes, leading to ECM accumulation [14]. The ability of FLU to restore redox homeostasis offers a significant therapeutic advantage, suggesting that S1R agonists can directly counteract a primary pathogenic factor in diabetic glaucoma and prevent TM damage.

While our findings strongly support S1R activation as an antifibrotic and protective strategy in diabetic models, certain limitations exist. The differential IOP response observed across the in vivo models highlights the complex, multifactorial nature of fibrotic changes and indicates that TM injury and fibrosis may precede the development of measurable ocular hypertension. Furthermore, the antifibrotic and protective effects were primarily established in vitro, and further in vivo studies are required to confirm the therapeutic efficacy of S1R activation fully.

## 4. Materials and Methods

### 4.1. Ethical Approval

Animal procedures were conducted in accordance with the regulations of the Committee on the Care and Use of Laboratory Animals of the Council on Animal Care at Semmelweis University, Budapest, Hungary (PEI/001/380-4/2013).

### 4.2. Materials

Standard plasticware was obtained from Sarstedt (Nümbrecht, Germany), and chemical reagents were purchased from Sigma-Aldrich/Merck (St. Louis, MO, USA), unless otherwise specified.

### 4.3. In Vivo Studies

#### 4.3.1. HFD/STZ Rat Model

Male Wistar rats at 8 weeks of age, weighing 250 ± 20 g, were purchased from Toxi-Coop Toxicological Research Centre (Dunakeszi, Hungary). The animals were kept under a 12 h light/dark cycle at a constant temperature of 22–24 °C, and housed in groups of three in plastic cages. Rodent chow and tap water were provided to the animals ad libitum. Analgesia was achieved with buprenorphine (Bupaq Multidose; Orion Pharma, Budapest, Hungary, 0.03 mg/bwkg), and appropriate anaesthesia using the combination of 75 mg/bwkg ketamine (Calypsol; Richter Gedeon, Budapest, Hungary; A31108) and 10 mg/bwkg xylazine (CP-Xylazin; Produlab pharma, Raamsdonksveer, The Netherlands) was used for all relevant procedures.

Prior to the beginning of the study, baseline blood glucose levels were measured. Subjects within normal glucose range were then randomly assigned to either the control (CTRL) or diabetic (high-fat diet/streptozotocin, HFD/STZ) group (10 animals/group). After one week of acclimatization rats in the diabetic group were given a HFD (Animalab, Vác, Hungary; Altromin, C 1090-45, fat content: 22%, energy from fat: 45%) that was maintained throughout the 35-week study period. Six weeks into the HFD, a single intraperitoneal (ip.) 35 mg/bwkg STZ (S0130) injection dissolved in 0.1 M citrate buffer (pH 4.5) was administered to accelerate the development of T2DM. The control group received standard rodent chow (ssniff R/M-Z + H 10 mm, Toxi-Coop, fat content: 9%) and an equivalent volume of citrate buffer without STZ. Fasting blood glucose levels were measured 72 h post-STZ from the tail vein using a D-Cont IDEAL glucose meter (77 Elektronika, Budapest, Hungary); animals with values above 15 mmol/L were considered diabetic. BW of the animals was monitored continuously.

IOP of the animals was measured weekly in both eyes at the same time each week using a tonometer (Icare Tonolab, Icare Finland Oy, Vantaa, Finland). Mean values of eight constitutive measurements were calculated.

At the end of the experimental period, the rats were anaesthetized using the combination of 75 mg/bwkg ketamine (Calypsol; Richter Gedeon; Budapest, Hungary, A31108) and 10 mg/bwkg xylazine (CP-Xylazin; Produlab pharma, Raamsdonksveer, The Netherlands), then sacrificed by drawing terminal blood. Eyes were enucleated, dissected, and fixed or immediately snap-frozen for further investigation.

#### 4.3.2. db/db Mouse Model

The BKS diabetic mouse strain (JAX stock #000642) was acquired from the Jackson Laboratory (Bar Harbor, ME, USA). The colony was maintained by pairing repulsion double heterozygotes (Dock7m +/+ Leprdb). For the experiments, adult male diabetic mice (Leprdb/Leprdb; referred to as db/db) and their misty littermates (Dock7m/Dock7m; controls) were selected. The mice were kept under a 12 h light/dark cycle at 22–24 °C, and housed in groups of five. Rodent chow and tap water were provided to the animals ad libitum. BW of the animals was measured, and blood glucose levels were assessed using a D-Cont IDEAL glucose meter (77 Elektronika).

At the end of the experimental period, the mice were anaesthetized using the combination of 75 mg/bwkg ketamine (Calypsol; Richter Gedeon; A31108) and 10 mg/bwkg xylazine (CP-Xylazin; Produlab pharma). IOP of the animals was measured with a tonometer (Icare Tonolab) in both eyes, and the mean of the eight constitutive measurements was calculated. The procedure of final sacrifice and ocular tissue preparation was the same as described above.

### 4.4. In Vitro Studies

#### 4.4.1. HTM5 Cell Culture

HTM5 cells were obtained from Abbot Clark (University of North Texas-Health Science Center, TX, USA) and provided by Xavier Gasull (University of Barcelona). Cells were cultured in Dulbecco’s modified Eagle’s medium (DMEM, 31885023, 10566016, Gibco, Thermo Fisher Scientific, Waltham, MA, USA) containing 5.5 mM glucose, 10% fetal bovine serum (FBS; 15070063, Thermo Fisher Scientific), and 1% penicillin/streptomycin (PenStrep, 15140122, Gibco), incubated in a humidified atmosphere (5% CO_2_, 37 °C). For glucose induction, the medium was replaced with DMEM containing 25 mM glucose alone (HG) or with 15 µM fluvoxamine maleate (F2802) (HG + FLU). The HG concentration was determined based on the methyl-thiazolyl diphenyl-tetrazolium bromide (MTT, M6494 Invitrogen, Carlsbad, CA, USA) glucose dose curve (Figure S2). The FLU concentration was selected based on preliminary dose–response MTT assays, in which 15 µM showed superior efficacy compared with lower doses while maintaining cell viability. Treatments were carried out in DMEM containing 1% FBS following a 4 h serum starvation. Cells grown in a 5.5 mM glucose medium (low glucose) served as the control group (LG).

#### 4.4.2. prTM Cell Culture

Seven days before termination, two Wistar rats from both groups (CTRL and HFD/STZ) were anaesthetised, and 10 µL of magnetic bead solution (PM-20-10, Spherotech, Lake Forest, IL, USA; SPHERO Polystyrene Magnetic Particles 2.5%) was injected into the anterior segment of one eye using a 25 µL Hamilton syringe. Before and after injection, a topical analgesic eye drop (6499318.00.00, Novesine 0.4%, OmniVision, Puchheim, Germany) was administered. During termination, the eyes were enucleated and then dissected under a light microscope. The globe was bisected by cutting beneath the limbus, and the anterior segments were separated.

The isolated tissue was transferred into a 5 mL Eppendorf tube for enzymatic digestion for two h at 37 °C. The digestion solution contained 2.5 U/mL dispase (CLS354235), 750 U/mL collagenase type II (17101-015, Gibco), and 950 µL Hank’s Balanced Salt Solution (HBSS, HBSS-1A, Capricorn Scientific, Ebsdorfergrund, Germany). The cell suspension was transferred into a 0.5% bovine serum albumin (BSA, 10500-064, Gibco)-2 mM EDTA (E-5134)–phosphate-buffered saline (PBS, 806552) solution, then centrifuged at 1800 rpm for 10 min. The resuspended cell pellet was subjected to a magnetic field to isolate the target cells (LS Columns, 130-042-401; MACS SmartStrainers, 130-098-463; Miltenyi Biotech, Bergisch Gladbach, Germany). The bead containing TM cells was eluted from the column with BSA-EDTA-PBS solution, then centrifuged (1800 rpm, 10 min). The cell pellet was resuspended and cultured in a 1:1 mixture of 5.5:25 mM (1:4.5 g/L) DMEM supplemented with 10% FBS, 1% PenStrep, 1× Amphotericin B (AMP-B, Capricorn) in a humidified atmosphere containing 5% CO_2_, at 37 °C.

TM cell identity was verified by a 7-day treatment with 100 nM dexamethasone (D0710000) followed by ICC targeting the steroid-inducible marker myocilin. Positive staining confirmed the successful isolation and lineage of the TM cells (Figure S1).

### 4.5. Histology

#### 4.5.1. Tissue Fixation and Paraffin Embedding

Following euthanasia, the rat eyes were dissected under a light microscope. The posterior segment of each eye was cut open, and the lenses were carefully removed. The remaining eye cups were filled with 4% paraformaldehyde (PFA, J61899.AK, Thermo Fisher Scientific) and immersed in PFA for 24 h at room temperature (RT) to ensure complete fixation. The tissues were then dehydrated through a graded series of ethanol and embedded in paraffin blocks. The eyes were sectioned, and 4 µm slides were deparaffinized in xylol (214736, 2 × 10 min) and were rehydrated in graded alcohol series.

#### 4.5.2. Picrosirius Red Staining

To visualize collagen deposition, slides prepared from rat anterior segments were stained with picrosirius red solution for 60 min, then washed in acidified distilled water. The slides were then dehydrated in graded alcohol series, mounted with coverslips, and digitized with a Panoramic1000 slide scanner (3DHistech, Budapest, Hungary).

#### 4.5.3. Immunohistochemical Staining for S1R

On the rat anterior segment slides, heat-induced epitope retrieval was performed in citrate buffer (pH 9, 30 min, 95 °C). Endogenous peroxidase activity was quenched with hydrogen peroxide solution (3.08027, Spectrum-3D, 3 *v*/*v*%, 15 min, RT). A specific epitope blocking was performed in 5% skim milk (70166) diluted in TRIS-buffered saline solution. Samples were incubated with an anti-S1R antibody (42-3300, Invitrogen, 1 h, RT), then a horseradish peroxidase (HRP)-conjugated secondary antibody (VC001, VisUCyte, R&D Systems, Hercules, CA, USA) was applied for 1 h at RT. A chromogenic reaction substrate (SK-4103-100, ImmPACT DAB EqV, Vector Laboratories, Inc., Newark, CA, USA) was utilized to visualize the primary antibody. Slides were stained with hematoxylin & eosin as described previously. Visualization was performed as described in Section 4.5.2.

### 4.6. Immunofluorescent Labeling

The mouse eye was bisected behind the limbus, the lens and vitreous were removed, and the iris was peeled back to expose and separate the TM. The tissue was subsequently fixed in 4% PFA for 24 h. The section was incubated overnight (ON) at 4 °C with S1R primary antibody (1:250, Invitrogen, 42-3300) followed by incubation with the specific secondary anti–rabbit Alexa Fluor 568 (A-11011, Invitrogen) conjugate. F-actin was labeled using a fluorescent phalloidin conjugate (1:1000; Alexa Fluor 546; A22283 Invitrogen) before nuclei were labeled with Hoechst (33342, Invitrogen, 5 μM, 10 min, RT). Coverslips were mounted using ProLong Anti-Fade (P36980, Thermo Fisher Scientific). Fluorescent images were acquired with an Abberior Expert Line confocal microscope (Abberior Instruments, Göttingen, Germany).

### 4.7. Immunocytochemistry (ICC)

HTM5 and prTM cells were seeded onto gelatin-coated glass-bottom culture chamber slides, utilizing 30,000 cells/well in 8-well chambers and 40,000 cells/well in 4-well chambers, respectively. HTM5 cells were induced with HG/HG + FLU medium for 48 h, prTM were incubated for 48 h in DMEM (1:1 mixture of 5.5:25 mM glucose) alone or supplemented with 15 µM FLU (HFD/STZ + FLU). Wells were then rinsed with PBS and fixed with 4% PFA. Fixation was followed by permeabilization with 0.1% Triton X-100 (X100) for 10 min at RT. ICC for S1R was performed on cells fixed with ice-cold methanol (322415) without subsequent permeabilization. Samples were blocked for 1 h at RT in a blocking buffer composed of 1% BSA and 10% goat serum (31873, Invitrogen) in PBS, containing 0.1% Tween-20 (93773). Cells were incubated ON at 4 °C with a primary antibody targeting rabbit anti-S1R (1:50, 423300, Invitrogen), rabbit anti-Fn (1:100; ab2413, abcam) or rabbit anti-myocilin (1:200, ab41552, abcam), followed by a 1 h incubation at RT with Alexa Fluor 568- and Alexa Fluor 488-conjugated chicken anti-rabbit secondary antibody (1:500, A110036, A21441, Invitrogen). F-actin was labeled by incubating the cells with Alexa Fluor 546-phalloidin (1:1000, A22283, Invitrogen) for 1 h at RT. Nuclear staining was performed using Hoechst (H3570, Invitrogen). Fluorescent images were obtained using a Nikon Eclipse Ti2 inverted fluorescence microscope, and image analysis was executed using Nikon AR software (version 5.21.03). Integrated density was calculated by multiplying the mean fluorescence intensity of the selected region of interest (ROI) by the area of the ROI.

### 4.8. Proliferation Assay

HTM5 cells were plated in a 96-well plate at a density of 8000 cells/well, and induced with HG/HG + FLU medium for 48 h. The cells were then incubated with MTT (5 μg/mL) for 3 h at 37 °C. The supernatant was collected, and a 100 µL mixture of ethanol (1.00971.2500) and dimethyl sulfoxide (DMSO, D4540, 1:1) was added to each well. Using the supernatant, LDH assay (C20300, Invitrogen) was performed to evaluate cell death based on the manufacturer’s protocol. Absorbance of the solubilized formazan crystals was detected at 570 nm (SpectroStar Nano microplate reader, BMG Labtech, Ortenberg, Germany).

### 4.9. Reverse Transcription-Quantitative Polymerase Chain Reaction (RT-qPCR)

HTM5 cells were plated in a 24-well plate at a density of 70,000 cells/well and induced with HG/HG + FLU medium for 48 h. Total RNA Isolation Mini Kit (RB300, Geneaid Biotech, New Taipei City, Taiwan) was used to isolate total RNA following the measurement of quality and quantity of the RNA on a NanoDrop One Spectrophotometer (Thermo Fisher, Wilmington, DE, USA). Using a First Strand cDNA Synthesis Kit for RT-PCR (K1642, Thermo Fisher Scientific), equal amounts of RNA were reverse-transcribed to complementary DNA. Quantification of *TGF-β2* and *COL1A1* mRNA levels was performed using 1 μL of cDNA in a qPCR reaction containing 10 μL of SYBR Green I Master Mix (04707516001, Roche Diagnostics, Basel, Switzerland) and 10 pmol/μL of each gene-specific primer (Invitrogen, primer pairs listed in Table 1). Data analysis was carried out with the LightCycler 480 software (version 1.5.0; Roche Diagnostics). The expression levels of target genes were normalized to 18S ribosomal RNA (RN18S) from the same cDNA samples as an internal control.

### 4.10. Western Blot

HTM5 cells were plated in a 6-well plate at a density of 300,000 cells/well, then incubated with HG or HG + FLU medium for 48 h. prTM cells derived from CTRL or HFD/STZ rats were seeded into a 6-well plate at a density of 120,000 cells/well and grown for 48 h in DMEM (1:1 mixture of 5.5:25 mM glucose) alone or with 15 μM FLU (HFD/STZ + FLU). The cells were detached by trypsinization (Trypsin-EDTA, 25200072, Thermo Scientific) and centrifuged at 2000 rpm, 5 min, at 4 °C. After washing with ice-cold PBS, cell pellets were lysed by adding freshly prepared lysis buffer composed of extraction buffer (FNN0011, Invitrogen), 2 µL/mL phenylmethanesulfonyl-fluoride (PMSF, P7626) and 10× protease inhibitor cocktail (P8340). The lysates were incubated on ice for 30 min, then complete cellular disruption and protein release were ensured by sonication (Fisher Scientific, Hampton, NH, USA). Cell debris was then removed (13,000× *g*, 10 min, 4 °C).

After determining the total protein concentration (Protein DC kit, 5000111, Bio-Rad Laboratories, Hercules, CA, USA), a total of 6–10 ng of protein was separated on a 4–20% gradient polyacrylamide gel (Mini-PROTEAN TGX Precast Gel, Bio-Rad), and subsequently transferred onto nitrocellulose membranes (Trans-Blot Turbo Mini 0.2 µm Nitrocellulose Transfer Packs, Bio-Rad). The membranes were blocked for 1 h at room temperature with either 5% skim milk or 5% BSA in TBS. After blocking, the membranes were incubated overnight at 4 °C with specific primary antibodies, including mouse anti-S1R (1:1000; sc-166392, SantaCruz Biotechnology, Dallas, TX, USA), rabbit anti-COL4A1 (1:500; Abcam, Cambridge, UK, ab6586), rabbit anti-Fn (1:5000; ab2413, Abcam), and rabbit anti-αSMA (1:2000; ab124964; Abcam). Following washing, membranes were incubated with HRP-linked secondary antibodies (anti-mouse: 7076S, 1:2000; anti-rabbit: 7074S, 1:3000; Cell Signaling Technology, Danvers, MA, USA).

Protein bands were visualized using chemiluminescent substrate (WBLUR, Immobilon Forte Western HRP substrate, Millipore, Burlington, MA, USA, or 34094, Femto Maximum Sensitivity Substrate, Thermo Scientific) and imaged using the ChemiDoc MP Imaging System (Bio-Rad). Densitometric analysis was performed using Image Lab software (version 6.1.0, Bio-Rad Laboratories), in which the integrated optical densities of the target bands were normalized against Ponceau S (A40000279, Thermo Fischer Scientific) staining.

### 4.11. Subcellular Fractionation

HTM5 cells were plated and treated as described above (Section 4.10). Subcellular fractionation was performed according to the manufacturer’s protocol using the Subcellular Protein Fractionation Kit (78840, Thermo Fisher Scientific). The resulting fractions were analyzed by Western blot to determine the cellular distribution of S1R.

### 4.12. NO Measurement

NO production was assessed using the fluorescent probe DAF-FM diacetate (D23844, Invitrogen). HTM5 cells were seeded in gelatin-coated 96-well black plate (Greiner Bio-One, Kremsmünster, Austria) at a density of 8000 cells/well, then exposed to HG or HG + FLU medium for 24 or 48 h. After treatment, cells were incubated with 5 μM DAF-FM, then NO levels were quantified using a ClarioStar microplate reader (BMG Labtech).

### 4.13. Detection of ROS

ROS generation was measured using CellRox Green Reagent (C10444, Thermo Fisher Scientific) assay. Cells were plated in a gelatin-coated 96-well black plate (Greiner) at a density of 8000 cells/well, then induced with HG or HG + FLU medium for 48 h. The plate was incubated with 5 µM CellROX Green Reagent diluted in HBSS for 30 min at 37 °C. Nuclear staining was performed using Hoechst. Cells were washed with HBSS, and mounting medium (50001, Ibidi, Gräfelfing, Germany) was added directly to the wells. Fluorescence was subsequently visualized using a Nikon Ti2 microscope. Images were analyzed using ImageJ (https://imagej.net/ij/, accessed on 9 December 2024, US National Institute of Health, Bethesda, MD, USA), by classifying signal intensities and denoting the base and elevated signal areas. Eight ROIs were analyzed, and the values were normalized to cell count, determined using DAPI nuclear staining.

### 4.14. Statistical Analysis

Data are presented as means ± standard deviation (SD). Prism software (version 10.1.0; GraphPad Software, San Diego, CA, USA) was used for statistical analysis. Data normality and homoscedasticity were assessed using the Shapiro–Wilk normality test. For datasets that passed the normality test and showed equal variance, parametric tests (One-way ANOVA with Holm–Sidak post hoc) were used. In cases where the data failed the normality test or showed significant heteroscedasticity, the non-parametric Kruskal–Wallis ANOVA on ranks followed by Dunn’s correction was applied. The statistically significant level was set at *p* < 0.05.

## 5. Conclusions

Our study showed that in vitro hyperglycaemia and in vivo DM induce significant fibrosis in the TM, demonstrated by excessive ECM accumulation and cytoskeletal remodeling, thereby contributing to increased outflow resistance. Crucially, the S1R agonist FLU successfully mitigated TM fibrosis, restored redox homeostasis, and elevated the levels of protective NO in HG-exposed HTM5 cells. These results strongly suggest that S1R activation may represent a new therapeutic target against hyperglycaemia-induced TM injury and DM-associated glaucoma.

## 6. Patents

US20190209575A1: “Novel use of sigma-1 receptor agonist compounds”. The invention describes new formulations and techniques for preventing fibrotic disease across multiple organs.

## Figures and Tables

**Table 1:** The primer pairs used for RT-qPCR.

Gene	NCBI ID		Primer Pairs	Product Length
*RN18S*	NR_003286.4	Forward:	5′ GGCGGCGACGACCCATTC 3′	136
		Reverse:	5′ TGGATGTGAGCCGTTTCTCAGG 3′	
*TGF-β2*	NM_003238.6	Forward:	5′ TACAACAGCACCAGGGACTT 3′	154
		Reverse:	5′ TAACAACTGGGCAGACGTTTCG 3′	
*COL1A1*	NM_053304.1	Forward:	5′ TCAAGATGTGCCACTCTGAC 3′	231
		Reverse:	5′ CATACTCGAACTGGAATCCA 3′	

## Data Availability

The original contributions presented in this study are included in the article/Supplementary Material. Further inquiries can be directed to the corresponding author.

## Supplementary Materials

The following supporting information can be downloaded at: https://www.mdpi.com/article/10.3390/ph19030385/s1. Figure S1. Primary rat trabecular meshwork (prTM) cells were isolated with the bead-injection method, confirmed by dexatmethasone (Dex)-induced myocilin expression. Figure S2. 25 mM glucose was chosen for high-glucose (HG)-induction of human trabecular meshwork (HTM5) cells.

## References

[1] Shaw J.E., Sicree R.A., Zimmet P.Z. (2010). Global estimates of the prevalence of diabetes for 2010 and 2030. Diabetes Res. Clin. Pract..

[2] Zhang P., Zhang X., Brown J., Vistisen D., Sicree R., Shaw J., Nichols G. (2010). Global healthcare expenditure on diabetes for 2010 and 2030. Diabetes Res. Clin. Pract..

[3] Weng J., Ross C., Baker J., Alfuraih S., Shamloo K., Sharma A. (2023). Diabetes-Associated Hyperglycemia Causes Rapid-Onset Ocular Surface Damage. Investig. Ophthalmol. Vis. Sci..

[4] Nentwich M.M., Ulbig M.W. (2015). Diabetic retinopathy—Ocular complications of diabetes mellitus. World J. Diabetes.

[5] Sayin N., Kara N., Pekel G. (2015). Ocular complications of diabetes mellitus. World J. Diabetes.

[6] Zhao D., Cho J., Kim M.H., Friedman D.S., Guallar E. (2015). Diabetes, fasting glucose, and the risk of glaucoma: A meta-analysis. Ophthalmology.

[7] Song B.J., Aiello L.P., Pasquale L.R. (2016). Presence and Risk Factors for Glaucoma in Patients with Diabetes. Curr. Diab Rep..

[8] Zhao Y.X., Chen X.W. (2017). Diabetes and risk of glaucoma: Systematic review and a Meta-analysis of prospective cohort studies. Int. J. Ophthalmol..

[9] Wong V.H., Bui B.V., Vingrys A.J. (2011). Clinical and experimental links between diabetes and glaucoma. Clin. Exp. Optom..

[10] Ren R., Zhang G.-W., Yang W.-Y., Xie Y., Wei M., Guan H.-J., Ji M. (2023). Concentrations of glucose metabolites in the aqueous humour of diabetic cataract eyes. Eur. J. Ophthalmol..

[11] Sato T., Roy S. (2002). Effect of High Glucose on Fibronectin Expression and Cell Proliferation in Trabecular Meshwork Cells. IOVS.

[12] Chen H.Y., Ko M.L., Chan H.L. (2024). Effects of hyperglycemia on the TGF-beta pathway in trabecular meshwork cells. Biochim. Biophys. Acta Gen. Subj..

[13] Pimentel L.G., Gracitelli C.P., da Silva L.S., Souza A.K., Prata T.S. (2015). Association between Glucose Levels and Intraocular Pressure: Pre- and Postprandial Analysis in Diabetic and Nondiabetic Patients. J. Ophthalmol..

[14] Singh S., Patel N.A., Soundararajan A., Pattabiraman P.P. (2024). High Glucose-Induced Transcriptomic Changes in Human Trabecular Meshwork Cells. Res. Sq..

[15] Wang M.Y., Liu W.J., Wu L.Y., Wang G., Zhang C.L., Liu J. (2023). The Research Progress in Transforming Growth Factor-beta2. Cells.

[16] Tran M.N., Medveczki T., Besztercei B., Torok G., Szabo A.J., Gasull X., Kovacs I., Fekete A., Hodrea J. (2023). Sigma-1 Receptor Activation Is Protective against TGFbeta2-Induced Extracellular Matrix Changes in Human Trabecular Meshwork Cells. Life.

[17] Fleenor D.L., Shepard A.R., Hellberg P.E., Jacobson N., Pang I.H., Clark A.F. (2006). TGFbeta2-induced changes in human trabecular meshwork: Implications for intraocular pressure. Investig. Ophthalmol. Vis. Sci..

[18] Sethi A., Jain A., Zode G.S., Wordinger R.J., Clark A.F. (2011). Role of TGFbeta/Smad signaling in gremlin induction of human trabecular meshwork extracellular matrix proteins. Investig. Ophthalmol. Vis. Sci..

[19] Mayorca-Guiliani A.E., Leeming D.J., Henriksen K., Mortensen J.H., Nielsen S.H., Anstee Q.M., Sanyal A.J., Karsdal M.A., Schuppan D. (2025). ECM formation and degradation during fibrosis, repair, and regeneration. NPJ Metab. Health Dis..

[20] Zhao W.J., Fan C.L., Hu X.M., Ban X.X., Wan H., He Y., Zhang Q., Xiong K. (2023). Regulated Cell Death of Retinal Ganglion Cells in Glaucoma: Molecular Insights and Therapeutic Potentials. Cell Mol. Neurobiol..

[21] Yao F., Peng J., Zhang E., Ji D., Gao Z., Tang Y., Yao X., Xia X. (2023). Pathologically high intraocular pressure disturbs normal iron homeostasis and leads to retinal ganglion cell ferroptosis in glaucoma. Cell Death Differ..

[22] Pang J.J., Frankfort B.J., Gross R.L., Wu S.M. (2015). Elevated intraocular pressure decreases response sensitivity of inner retinal neurons in experimental glaucoma mice. Proc. Natl. Acad. Sci. USA.

[23] Lee H.P., Tsung T.H., Tsai Y.C., Chen Y.H., Lu D.W. (2024). Glaucoma: Current and New Therapeutic Approaches. Biomedicines.

[24] Albayrak Y., Hashimoto K. (2017). Sigma-1 Receptor Agonists and Their Clinical Implications in Neuropsychiatric Disorders. Adv. Exp. Med. Biol..

[25] Ryskamp D.A., Korban S., Zhemkov V., Kraskovskaya N., Bezprozvanny I. (2019). Neuronal Sigma-1 Receptors: Signaling Functions and Protective Roles in Neurodegenerative Diseases. Front. Neurosci..

[26] Schoenwald R.D., Barfknecht C.F., Xia A., Newton R.E. (1993). The Presence of cr-Receptors in the Lacrimal Gland. J. Ocul. Pharmacol..

[27] Bucolo C., Campana G., Di Toro R., Cacciaguerra S., Spampinato S. (1999). ς1 Recognition Sites in Rabbit Iris-Ciliary Body: Topical ς1-Site Agonists Lower Intraocular Pressure. J. Pharmacol. Exp. Ther..

[28] Wang L., Duncan G. (2006). Silencing of sigma-1 receptor induces cell death in human lens cells. Exp. Cell Res..

[29] Xu Z., Lei Y., Qin H., Zhang S., Li P., Yao K. (2022). Sigma-1 Receptor in Retina: Neuroprotective Effects and Potential Mechanisms. Int. J. Mol. Sci..

[30] Senda T., Matsuno K., Mita S. (1997). The Presence of σ Receptor Subtypes in Bovine Retinal Membranes. Exp. Eye Res..

[31] Meng B., Li H., Sun X., Qu W., Yang B., Cheng F., Shi L., Yuan H. (2017). sigma-1 receptor stimulation protects against pressure-induced damage through InsR-MAPK signaling in human trabecular meshwork cells. Mol. Med. Rep..

[32] Ke H., Su X., Dong C., He Z., Song Q., Song C., Zhou J., Liao W., Wang C., Yang S. (2024). Sigma-1 receptor exerts protective effects on ameliorating nephrolithiasis by modulating endoplasmic reticulum-mitochondrion association and inhibiting endoplasmic reticulum stress-induced apoptosis in renal tubular epithelial cells. Redox Rep..

[33] Zhao X., Zhu L., Liu D., Chi T., Ji X., Liu P., Yang X., Tian X., Zou L. (2019). Sigma-1 receptor protects against endoplasmic reticulum stress-mediated apoptosis in mice with cerebral ischemia/reperfusion injury. Apoptosis.

[34] Morihara R., Yamashita T., Liu X., Nakano Y., Fukui Y., Sato K., Ohta Y., Hishikawa N., Shang J., Abe K. (2018). Protective effect of a novel sigma-1 receptor agonist is associated with reduced endoplasmic reticulum stress in stroke male mice. J. Neurosci. Res..

[35] Tejada M.A., Montilla-Garcia A., Sanchez-Fernandez C., Entrena J.M., Perazzoli G., Baeyens J.M., Cobos E.J. (2014). Sigma-1 receptor inhibition reverses acute inflammatory hyperalgesia in mice: Role of peripheral sigma-1 receptors. Psychopharmacology.

[36] Rosen D.A., Seki S.M., Fernández-Castañeda A., Beiter R.M., Eccles J.D., Woodfolk J.A., Gaultier A. (2019). Modulation of the sigma-1 receptor–IRE1 pathway is beneficial in preclinical models of inflammation and sepsis. Sci. Transl. Med..

[37] Li Z., Zhou J., Cui S., Hu S., Li B., Liu X., Zhang C., Zou Y., Hu Y., Yu Y. (2024). Activation of sigma-1 receptor ameliorates sepsis-induced myocardial injury by mediating the Nrf2/HO1 signaling pathway to attenuate mitochondrial oxidative stress. Int. Immunopharmacol..

[38] Pal A., Fontanilla D., Gopalakrishnan A., Chae Y.K., Markley J.L., Ruoho A.E. (2012). The sigma-1 receptor protects against cellular oxidative stress and activates antioxidant response elements. Eur. J. Pharmacol..

[39] Hodrea J., Tran M.N., Besztercei B., Medveczki T., Szabo A.J., Orfi L., Kovacs I., Fekete A. (2023). Sigma-1 Receptor Agonist Fluvoxamine Ameliorates Fibrotic Response of Trabecular Meshwork Cells. Int. J. Mol. Sci..

[40] Balogh D.B., Hodrea J., Saeed A., Cserhalmi M., Rozsahegyi A., Lakat T., Lenart L., Szabo A.J., Wagner L.J., Fekete A. (2024). Sigma-1 Receptor as a Novel Therapeutic Target in Diabetic Kidney Disease. Int. J. Mol. Sci..

[41] Dismuke W.M., Mbadugha C.C., Ellis D.Z. (2008). NO-induced regulation of human trabecular meshwork cell volume and aqueous humor outflow facility involve the BKCa ion channel. Am. J. Physiol. Cell Physiol..

[42] Chen H.Y., Chou H.C., Ho Y.J., Chang S.J., Liao E.C., Wei Y.S., Lin M.W., Wang Y.S., Chien Y.A., Yu X.R. (2021). Characterization of TGF-beta by Induced Oxidative Stress in Human Trabecular Meshwork Cells. Antioxidants.

[43] Friedlander M. (2007). Fibrosis and diseases of the eye. J. Clin. Investig..

[44] Vranka J.A., Kelley M.J., Acott T.S., Keller K.E. (2015). Extracellular matrix in the trabecular meshwork: Intraocular pressure regulation and dysregulation in glaucoma. Exp. Eye Res..

[45] Davies P., Duncan G., Pynsent P.B., Arber A.L., Lucas V.A. (1984). Aqueous Humour Glucose Concentration in Cataract Patients and its Effects on the Lens. Exe. Eye Res..

[46] Bermudez J.Y., Montecchi-Palmer M., Mao W., Clark A.F. (2017). Cross-linked actin networks (CLANs) in glaucoma. Exp. Eye Res..

[47] Sit A.J. (2014). Intraocular pressure variations: Causes and clinical significance. Can. J. Ophthalmol..

[48] Schmidl D., Schmetterer L., Garhofer G., Popa-Cherecheanu A. (2015). Pharmacotherapy of glaucoma. J. Ocul. Pharmacol. Ther..

[49] (2017). European Glaucoma Society Terminology and Guidelines for Glaucoma, 4th Edition—Chapter 2: Classification and terminology: Supported by the EGS Foundation. Br. J. Ophthalmol..

[50] Su T.P., Su T.C., Nakamura Y., Tsai S.Y. (2016). The Sigma-1 Receptor as a Pluripotent Modulator in Living Systems. Trends Pharmacol. Sci..

[51] Aishwarya R., Abdullah C.S., Morshed M., Remex N.S., Bhuiyan M.S. (2021). Sigmar1’s Molecular, Cellular, and Biological Functions in Regulating Cellular Pathophysiology. Front. Physiol..

[52] Du M., Jiang T., He S., Cheng B., Zhang X., Li L., Yang L., Gao W., Li Y., Wang Q. (2023). Sigma-1 Receptor as a Protective Factor for Diabetes-Associated Cognitive Dysfunction via Regulating Astrocytic Endoplasmic Reticulum-Mitochondrion Contact and Endoplasmic Reticulum Stress. Cells.

[53] Sukhatme V.P., Reiersen A.M., Vayttaden S.J., Sukhatme V.V. (2021). Fluvoxamine: A Review of Its Mechanism of Action and Its Role in COVID-19. Front. Pharmacol..

[54] Hindmarch I., Hashimoto K. (2010). Cognition and depression: The effects of fluvoxamine, a sigma-1 receptor agonist, reconsidered. Hum. Psychopharmacol..

[55] Mallone F., Costi R., Marenco M., Plateroti R., Minni A., Attanasio G., Artico M., Lambiase A. (2021). Understanding Drivers of Ocular Fibrosis: Current and Future Therapeutic Perspectives. Int. J. Mol. Sci..

[56] Tripathi R.C., Li J., Chan W.F.A., Tripathi B.J. (1994). Aqueous Humor in Glaucomatous Eyes Contains in Increased Level of TGF-beta2. Exp. Eye Res..

[57] Stamer W.D., Clark A.F. (2017). The many faces of the trabecular meshwork cell. Exp. Eye Res..

[58] Chakravarthy U., Hayes R.G., Stitt A.W., McAuley E., Arche D.B. (1998). Constitutive Nitric Oxide Synthase Expression in Retinal Vascular Endothelial Cells Is Suppressed by High Glucose and Advanced Glycation End Products. Diabetes.

[59] Park C.H., Kim J.W. (2012). Effect of advanced glycation end products on oxidative stress and senescence of trabecular meshwork cells. Korean J. Ophthalmol..

[60] Nathanson J.A., McKee M. (1995). Alterations of Ocular Nitric Oxide Synthase in Human Glaucoma. Investig. Ophthalmol. Vis. Sci..

[61] Doganay S., Evereklioglu C., Turkoz Y., Er H. (2002). Decreased nitric oxide production in primary open-angle glaucoma. Eur. J. Ophthalmol..

[62] Hayashi T. (2015). Conversion of psychological stress into cellular stress response: Roles of the sigma-1 receptor in the process. Psychiatry Clin. Neurosci..

[63] Saccà S.C., Pascotto A., Camicione P., Capris P., Izzotti A. (2005). Oxidative DNA Damage in the Human Trabecular Meshwork: Clinical Correlation in Patients with Primary Open-Angle Glaucoma. Arch. Ophthalmol..

[64] Zhou L., Li Y., Yue B.Y.J.T. (1999). Oxidative stress affects cytoskeletal structure and cell-matrix interactions in cells from an ocular tissue: The trabecular meshwork. J. Cell. Physiol..

