# Supplementary material for: The Sigma-1 Receptor Agonist Fluvoxamine Is Protective in Hyperglycaemia-Induced Dysfunction of Trabecular Meshwork Cells
Source: Pharmaceuticals (Basel). 2026 Feb 27;19(3):385. doi: 10.3390/ph19030385 (PMC13029636; doi:10.3390/ph19030385)
Supplement: Supplementary file 1 [file pharmaceuticals-19-00385-s001.zip › pharmaceuticals-4067889-supplementary.pdf]

Supplementary Materials

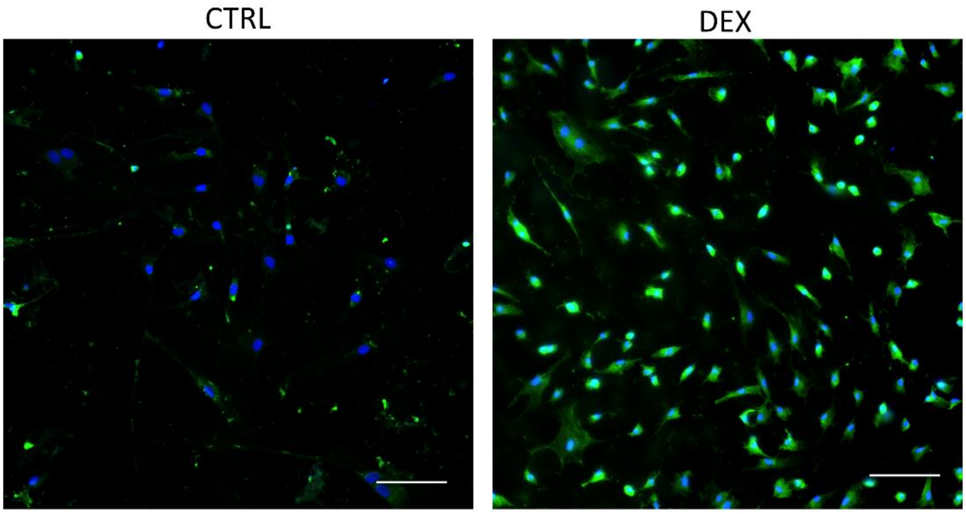

Figure S1. Primary rat trabecular meshwork (prTM) cells were isolated with the bead-injection method, confirmed by dexatmethasone (Dex)-induced myocilin expression. Representative images of Dex induced prTM cells. Cells exhibited strong myocilin expression after 7 days of 100 nM Dex treatment compared to non-treated cells (CTRL), confirming the trabecular meshwork (TM) identity of the isolated primary cells (myocilin: green, nuclei: blue; Nikon Eclipse Ti2 microscope, objective: 20 x; scale bar: 100 µm).

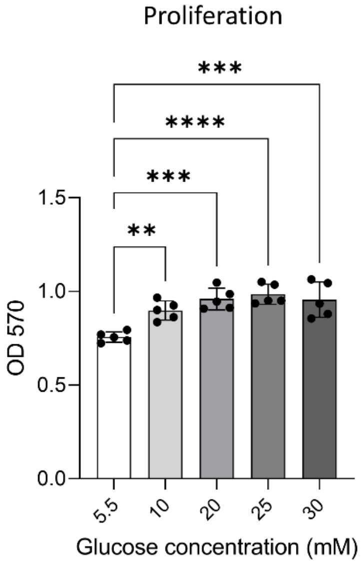

Figure S2. 25 mM glucose was chosen for high-glucose (HG)-induction of human trabecular meshwork (HTM5) cells. In order to determine the lowest concentration of glucose needed for the maximum effect in TM proliferation, we used the MTT assay. HTM5 cells were induced with 5.5-30 mM glucose for 48 h. Dose-dependent proliferation rate peaked at 25 mM. Therefore, 25 mM was selected as the optimal concentration for HG-induction, as it induced the highest proliferative response among the glucose concentrations tested and remained within physiologically relevant levels for diabetic AH. Bars indicate mean +/- SD; ** $p < 0.01$; *** $p < 0.001$; **** $p < 0.0001$; n = 5/group.
